# Essential workers' pandemic mobilities and the changing meanings of the commute

**DOI:** 10.1111/geoj.12447

**Published:** 2022-05-24

**Authors:** Anna Plyushteva

**Affiliations:** ^1^ Transport Studies Unit, School of Geography and the Environment University of Oxford Oxford UK

**Keywords:** commuting, COVID‐19, essential workers, mobility, pandemic, transport geography

## Abstract

This commentary reflects on the pandemic commute and its significance for, on one hand, engaging with the problematic category of essential work, and on the other, future geographical research on transport and mobilities. Drawing on essential workers' contributions to the ‘Not working from home’ public engagement project, I outline some experiences of commuting during the COVID‐19 pandemic. I illustrate the role of pandemic commuting in defining, and wrestling with, what the category of essential work might mean. I then discuss the ways in which attending to pandemic commutes may extend and reshape existing research on unequal mobilities. Some of the future research directions made more urgent by a focus on pandemic commutes include critical engagements with: first, intersectional inequalities in the journey to work; second, the category of ‘essential journeys’ as used in transport policy and practice; third, the positionality of academic researchers who work on the topic of commuting; and finally, the treatment of commuting time as an integral part of working time.

## Abstract 2

Essential workers' commutes during the COVID‐19 pandemic presented new challenges and amplified existing ones. The Not Working from Home public engagement project documented some of them. Pandemic commutes should shape future research on commuting and inequality within transport geography.

## 1 PANDEMIC IM/MOBILITIES

In my role as a qualitative transport geographer, I have been conducting research on different aspects of commuting for the last ten years. I have often felt exasperated by the view of the commute as a banal, repetitive, universally recognisable experience, the very definition of routine (for an alternative view, see, for example, Bissell, 2016). Similarly, I have aimed to problematise the notion, prevalent in much transport policy and practice, of the journey to work as the definitive *essential* trip, to be prioritised over other everyday mobilities (Plyushteva, 2019). As a theoretical and methodological challenge, the commute opens endless possibilities for nuanced engagements with geographic themes from gender relations to infrastructures, and from climate change to inequality (Jirón & Imilan, 2015). And yet, I did not anticipate that it would take a pandemic to make visible to a wider audience the profound ways in which the commute can divide, differentiate and dis/empower (for a related argument on the COVID19 pandemic and the visibility of borders, see Yip, 2021).

During the pandemic, everyday mobility and immobility have come into stark view. However, this renewed media and scholarly attention has mostly produced stories of interruption and stillness, an alleged sudden shift from ubiquitous mobility to near‐full immobility (see, for instance, Rutz et al., 2020; Searle et al., 2021). As many countries worldwide introduced extended lockdowns to curtail the spread of the novel coronavirus, attention shifted largely to the challenges of working from home. However, many others, and particularly those designated as ‘key’ or ‘essential’ workers, among whom many are in low‐paid and/or precarious employment, continued to make commuter journeys (Black et al., 2020; Lee et al., 2021).

The label of ‘essential work’ has inevitably been challenged for the inequities and injustices it can obscure, from the process of labelling itself, which some essential workers experienced as coercive and unilateral, to the wide range of working and mobility circumstances subsumed by this single category. From the perspective of geographic research on transport and mobilities, much work remains to be done on documenting and analysing essential workers' diverse experiences during COVID‐19. Furthermore, understanding pandemic commutes may open up new critical directions for researching the commute and its social impacts also beyond the COVID‐19 pandemic. In this commentary, I draw on the contributions submitted as part of a public engagement project entitled ‘Not working from home’ (https://www.notworkingfromhome.org/), in order to discuss, first, the impacts on essential workers of sustaining and modifying their commutes during the pandemic, and second, the wider implications for transport research on commuting. My principal argument is that research on the journey to work requires fundamental rethinking in light of the divisions between (to simplify) ‘the commuters’ and ‘the others’ created by the pandemic.

## 2 NOT WORKING FROM HOME

Immediately before the pandemic, transport planners and users in many locales worldwide would have defined the journey to work as an *essential* trip, regardless of the nature of work involved (see also Salazar, 2021, for a discussion of essential mobilities in historical context). However, the meaning of essential journeys changed during the course of the COVID‐19 pandemic. Since March 2020, travelling to a place of work has become a key characteristic not of all, or almost all, work, but specifically of *essential work*.

The ‘Not working from home’ project was a public engagement project which sought contributions from essential workers for an online exhibition of photos, short video recordings, and text, in order to document daily life during the pandemic for those who continued to commute. The project was motivated by my growing frustration, particularly during 2020, of a lack of engagement in news and social media with the lived experiences of essential workers (to be distinguished from public celebrations of their heroism and self‐sacrifice, be they cynical or heartfelt; see Yuill, 2022).

The designation of essential, critical, frontline or key, workers shifted over time and was geographically specific, but broadly included those employed in sectors such as healthcare, social care, education and childcare, food and transport, among others. While the contributions made by essential workers in sustaining life during the pandemic was publicly celebrated, the everyday challenges and joys such work entailed were made invisible by a public discourse dominated by concerns with immobility. As the world of the COVID‐19 pandemic was increasingly being construed as a world that stands still (an ‘Anthropause’, Rutz et al., 2020), experiences that did not comply with this overarching narrative, including the pandemic commute, were sidelined. If human relations were now allegedly defined by digitally mediated, home‐bound, increasingly claustrophobic interactions, how could we (those trapped/tranquil at home) make sense of, document and be moved by the everyday social and material encounters of pandemic commuting? I was keen to document the mobilities of essential workers on the participants' own terms, without framing their commutes as the exception to a general state of lockdown and stillness. In other words, the project was intended as a space in which workers themselves could document what ‘not working from home’ was, and not simply what it was not. The project gathered contributions between March and June 2021, and collected 70 stories, mostly from UK‐based participants.

## 3 ESSENTIAL WORKERS' COMMUTES

I was going to work only every other week.^1^ And for the first time, at the end of the month, I wasn't having to check the bank account all the time and think this much for the kids, this much for that … For the first time, there was money there. Not only because of the cost of the bus – we were also not going out, not eating out. But also because of the cost of the bus. (Mark, teacher based in Bristol, UK)

The designation of work as essential, and the need for many of those performing it to continue to travel to work during a pandemic, have been associated with profound health, financial and emotional implications. In a follow‐up research interview, Mark, a secondary school teacher, painted a vivid account of the economic implications of not working from home during COVID‐19 lockdowns. Even working remotely only half of the time resulted in a palpable difference in terms of the household budget. It meant having more money to cover the needs of Mark's children, but it also meant an easing of familiar money pressures, especially in the run‐up to payday. As a result, Mark empathised both with those working from home, and those who had not worked remotely at all. In follow‐up interviews, participants in the ‘Not working from home’ project often spoke of the immense toll of facing uncertainty, taking daily risks and baring responsibility. While these challenges have received some coverage in relation to ‘frontline’ places of work, the themes of fear, uncertainty and caring for others were also key in accounts of pandemic commuting. These could be glimpsed in narratives of walking down shuttered high streets, exchanging nervous looks following coughs and sneezes on public transport, or having to re‐learn to drive after years of riding the bus. Crucially, some participants felt that stopping commuting brought to those working from home immediate and often substantial monetary benefits. This was contrasted with the limited, or non‐existent, additional compensation for essential workers. Apart from additional (for potentially safer car and taxi journeys) or unreduced transport costs, for many participants sustaining a pandemic commute involved navigating new, frequently changing, reduced and/or unreliable public transport schedules and routes; experiencing fear in previously busy but now deserted transport and urban spaces; and enduring face coverings and continuous hand sanitising to a much greater extent than workers at home. For some, pandemic commutes also brought about the novel pleasure of a limitless supply of free seats on trains and buses.

For the majority of people (not all) who have spoken to me about commuting over the last decade, the journeys to and from work have always been something to endure. However, the commute's familiarity across geographies, socio‐economic groups and occupations could also bring about unexpected solidarities and connections. Discussing the trials of commuting could establish common ground where there previously was none, or fuel a conversation when few topics are appropriate. Pandemic commuting became both an extension and amplification of these contrasting dynamics. For some, it became the object of new camaraderie among essential workers in specific sectors, or even across sectoral divisions. It also created new lines of separation, for instance where employees in the same hospital could work from home some of the time, or not, depending on whether they had a clinical or administrative role. For many, the commute became a trial of endurance more than ever before, even when their ‘before’ commute had already felt barely manageable. With distinctive social, economic and emotional consequences compared to previous iterations of everyday mobility, navigating pandemic commuting also made visible new kinds of differences, not limited to those who did commute versus those who did not (for example, with night public transport services axed, a costly taxi ride was the only option for many night‐time pandemic commuters). Thus, a range of diverse constraints, capacities and negotiations could be discerned among the broader group of commuting essential workers, and many of those felt new and unprecedented. This included, for instance, identifying with those who *could* commute (as opposed to being stranded at home), with those who *had no choice but to* commute (instead of being free to be at home), or with those whose circumstances were too complex or variable to be captured in such simple binaries. While it is important to attend to these nuances with the utmost care, it is equally crucial to emphasise that none of the participants felt that continuing to commute during the pandemic was a straightforward matter of carrying on as before.

In the account presented here, I have aimed to steer away from notions of working from home as inherently good, safe or liberating, and not working from home as its opposite. Equally, I have sought to avoid implicitly or explicitly attaching such ideas to the category of the essential worker. While some of the participants in the ‘Not working from home’ project challenged externally imposed aspects of the essential worker designation (including sentimental expressions of gratitude, expectations of heroism and poor recognition and remuneration; Wood and Skeggs, 2020), it would be erroneous to assume the label was comprehensively rejected. It did, however, become something many participants wrestled with, as they considered the meanings and emotions of being called *essential* during pandemic times. Making sense of one's pandemic commute was a prominent aspect of this, leading me to argue that the non‐optional and non‐flexible commute became a definition of essential work in its own right. This is because essential workers, having to undertake a journey to work during lockdowns, first, continued to commute when others did not, and second, experienced daily journeys which were both a continuation of their pre‐pandemic commutes, and a new type of experience altogether, involving new precautions, risks, rhythms and interactions. The central role played by the commute in differentiating some types of pandemic work from others has prompted me to reflect more broadly on what research on commuting may look like beyond the COVID‐19 pandemic.

## 4 CHANGING MEANINGS OF THE COMMUTE

Commuting, in the aftermath of the COVID‐19 pandemic, has acquired new and changing meanings. It has acquired additional lines of differentiation; additional labels, categories and inequities; and new questions around the social and cultural dimensions of everyday mobility. Their enduring impact can be glimpsed in an anecdote shared by one transport sector professional. According to them, since the start of the pandemic, school leavers in the UK are not only asked what job they want to do by their teachers and carers, but specifically quizzed on whether they might want to prioritise the kind of job which allows remote working.^2^

In the interdisciplinary field of transport and mobility research, in which human geographers play a key role, the commute has long been seen as more than a routine, instrumental movement from place A to place B and back again. However, research on commuting inequalities has largely remained focused on unequal access to destinations, in particular to employment opportunities, and has often stood separate from work on the everyday journey as an uneven experience in its own right, with its practices, meanings, socialities and infrastructures (for further discussion, see Verlinghieri & Schwanen, 2020,). The pandemic commute problematises further these uneasy silos. Having never been equal or equalising, during the COVID‐19 pandemic commuting was particularly likely to become an economic and health penalty on many of the less well‐off, and disproportionately on Black, Asian and minority ethnic workers (Reuschke & Felstead, 2020; Smith et al., 2021).

The injustices of pandemic commuting seem to me to necessitate new research directions, particularly at the interface between the geography of transport and mobility, and other geographic sub‐disciplines. Intersectional understandings of pandemic commutes seem a key starting point, as the overrepresentation of intersectional disadvantaged groups in the essential workers category has not received sufficient attention in transport and mobility research. The relationship between long, uncomfortable, unpredictable, dangerous and/or expensive commutes on one hand, and precarious and/or poorly paid jobs on the other, has required greater attention for some time. However, the COVID‐19 pandemic has set it in ever‐starker terms, against a background of an increasingly optional and flexible white‐collar commute. Another set of questions that pandemic commutes bring starkly into view (and feminist transport and mobility geographers have explored for some time) is the problematic separation of the journey to work into its own, highly valued, essential or ‘non‐discretionary’ category, despite its profound entanglements with the equally essential mobilities of unpaid care (Plyushteva & Schwanen 2018; Ravensbergen et al., 2020; Sanchez de Madariaga & Zucchini 2019). Rethinking the relationship between the commute and essential‐ness in transport policy and research is more than a matter of terminology; it has far‐reaching implications for how travel time is valued, and thus for the ways in which transport infrastructure projects are planned and prioritised (Levin & Faith‐Ell, 2019).

Thirdly, the need for reflexivity among commuting researchers has never been greater, as for many of us the routine of commuting acquired distant, even foreign, qualities while working from home during COVID‐19 lockdowns. While existing research on commuting has often taken the experience of particular groups (white‐collar, relatively better‐off) as broadly representative of the journey to work, the pandemic commute problematises such universalising approaches, and calls for open conversations on positionality. Finally, pandemic commutes present a new angle on critical debates about the extent to which commuting can be considered part of work itself, and the time spent commuting, as part of the working day. These debates have significant implications for both workers and employers, for example in relation to remuneration, working hours and taxation. While different countries currently take different approaches to this (de Blécourt & Schoevaars, 2016), the growing gap between flexible and non‐flexible commuting arrangements, and the ways in which it maps onto inequalities of income and job security, should reinvigorate debates on how the costs of commuting are distributed among individuals, employers and the state.

In this commentary, I have drawn on what I learnt about pandemic commutes while conducting a public engagement project entitled ‘Not working from home.’ Reflecting on the contributions participants made to this project, I have suggested that understanding the pandemic commute as a defining characteristic of *essential* work can be a useful starting point for thinking about this problematic, and for some coercive, designation. In addition, I have reflected on the ways in which the everyday mobilities of essential workers during the COVID‐19 pandemic might inform future directions in geographic research on commuting, as part of a growing body of work on unequal mobilities.

## Data Availability

Data are available on request due to privacy/ethical restrictions.

## Endnotes

^1^
In the school where this participant worked, as vulnerable children and the children of essential workers continued to attend in person, teachers took turns to come into work every other week.

^2^
Representatives from several UK‐based non‐profit organisations who work in the transport and mobility sector were consulted as part of this project. Their contributions are gratefully acknowledged, but their comments are kept anonymous in any resulting publications.
